# The prevalence of psychological distress during pregnancy in Miyagi Prefecture for 3 years after the Great Eas t Japan Earthquake

**DOI:** 10.1186/s12199-021-00944-2

**Published:** 2021-02-26

**Authors:** Kaou Tanoue, Zen Watanabe, Hidekazu Nishigori, Noriyuki Iwama, Michihiro Satoh, Takahisa Murakami, Kousuke Tanaka, Satomi Sasaki, Kasumi Sakurai, Mami Ishikuro, Taku Obara, Masatoshi Saito, Junichi Sugawara, Nozomi Tatsuta, Shinichi Kuriyama, Takahiro Arima, Kunihiko Nakai, Nobuo Yaegashi, Hirohito Metoki, Japan Environment and Children’s Study (JECS) Group

**Affiliations:** 1 Department of Obstetrics and Gynecology, Tohoku University Graduate School of Medicine, 1-1, Seiryo-machi, Aoba-ku, Sendai, Miyagi 980-8574 Japan; 2 Environment and Genome Research Center, Tohoku University Graduate School of Medicine, 2-1 Seiryo-machi, Aoba-ku, Sendai, Miyagi 980-8573 Japan; 3 Fukushima Medical Center for Children and Women, Fukushima Medical University, 1 Hikariga-oka, Fukushima, 960-1295 Japan; 4 Division of Public Health, Hygiene and Epidemiology, Tohoku Medical Pharmaceutical University, 1-15-1 Fukumuro, Sendai, Miyagi 983-8536 Japan; 5 Department of Obstetrics and Gynecology, Hachinohe City Hospital, 3-1-1, Tamukai, Hachinohe, Aomori, 031-8555 Japan; 6 Tohoku Medical Megabank Organization, Tohoku University, 2-1 Seiryo-machi, Aoba-ku, Sendai, Miyagi 980-8573 Japan; 7 Department of Pharmaceutical Sciences, Tohoku University Hospital, 1-1 Seiryo-machi, Aoba-ku, Sendai, Miyagi 980-8574 Japan

**Keywords:** Psychological distress, Negative life events, Earthquake, Tsunami, Pregnant women

## Abstract

**Background:**

To examine changes in psychological distress prevalence among pregnant women in Miyagi Prefecture, which was directly affected by the Great East Japan Earthquake and tsunami, and compare it with the other, less damaged areas of Japan.

**Methods:**

This study was conducted in conjunction with the Japan Environment and Children`s Study. We examined 76,152 pregnant women including 8270 in Miyagi Regional Center and 67,882 in 13 other regional centers from the all-birth fixed data of the Japan Environment and Children’s Study. We then compared the prevalence and risk of distress in women in Miyagi Regional Center and women in the 13 regional centers for 3 years after the disaster.

**Results:**

Women in the Miyagi Regional Center suffered more psychological distress than those in the 13 regional centers: OR 1.38 (95% CI, 1.03–1.87) to 1.92 (95% CI, 1.42–2.60). Additionally, women in the *inland area* had a consistently higher prevalence of psychological distress compared to those from the 13 regional centers: OR 1.67 (95% CI, 1.18–2.38) to 2.19 (95% CI, 1.60–2.99).

**Conclusions:**

The lack of pre-disaster data in the Japan Environment and Children’s Study made it impossible to compare the incidence of psychological distress before and after the March 2011 Great East Japan Earthquake. However, 3 years after the Great East Japan Earthquake, the prevalence of pregnant women with psychological distress did not improve in Miyagi Regional Center. Further, the prevalence of mental illness in inland areas was consistently higher than that in the 13 regional centers after the disaster.

## Background

On 11 March 2011, a massive earthquake measuring 9.0 on the Richter scale struck northeast Japan and led to severe damage to the east coast of Japan. The Great East Japan Earthquake and subsequent tsunami resulted in 22,000 dead or missing, and approximately 400,000 houses collapsed [1]. Miyagi Prefecture is located on the coast of eastern Japan and was one of areas most affected by the disaster. In Miyagi Prefecture, 10,565 and 1220 people were dead or missing, respectively, and 238,119 houses were completely or partially destroyed [2]. This was Japan’s largest earthquake and the fourth largest in the world since 1900, according to the United States Geological Survey [3].

Previous studies have addressed the fact that devastation caused by a natural disaster affects maternal mental health, including perinatal depression [4, 5]. Perinatal depression is associated with women’s postnatal health and may impact not only newborn infants’ quality of care but also their subsequent growth and development [6–9]. O’Connor et al. [10] found that pregnant women’s anxiety levels at 32 weeks of pregnancy were closely related to behavioral and emotional disorders (overactivity, emotional disorders, relationship disorders) of 81-month-old infants. Thus, the mental impact on post-natal babies is more closely related to anxiety during pregnancy than postpartum anxiety and depression. In addition, fetal exposure to long-term maternal cortisol, which is over-secreted due to stress, may be a contributing factor to the mental impact on post-natal babies [10].

In Europe and the USA, suicide associated with mental illness, including postpartum depression, is a major cause of death in postpartum women, but the actual situation in Japan has not been fully studied. There were 63 cases of suicides among pregnant women in 23 wards of Tokyo from 2005 to 2014 (23 cases during pregnancy, 40 cases less than 1 year postpartum). The rate of suicides during pregnancy was more than twice the maternal mortality rate due to obstetric abnormalities in Tokyo [11]. The incidence rates of the onset of DSM-III-R major depressive episodes during pregnancy and within 3 months after delivery in Japan have been reported to be 5.6% and 5.0%, respectively [12]. Further, an unstable environment after a large-scale disaster might cause mental health problems; thus, attention should be paid to mental health care for pregnant women.

**Fig. 1:**
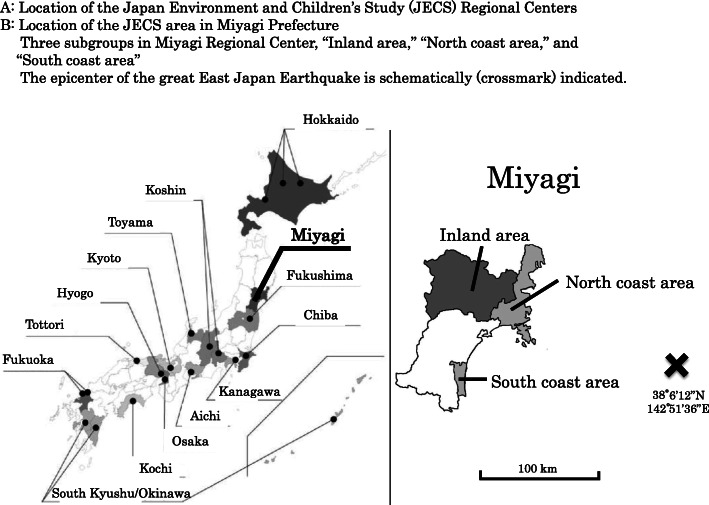
Location of 15 Regional Centers in Japan and location of the JECS area in Miyagi Prefecture for the Japan Environmental and Children's Study

As far as we know, there are no reports on the changes in the prevalence of psychological distress among pregnant women following large scale disasters. Nevertheless, Watanabe et al. [13] reported that the prevalence of pregnant women with psychological distress was high in Miyagi Prefecture after the Great East Japan Earthquake. The Ministry of the Environment launched a large-scale cohort epidemiological research project entitled the Japan Environment and Children’s Study (JECS) in January 2011. The target cohort included 100,000 children and their parents, and the purpose of the study was to investigate the association between environmental factors and children’s health and development [14]. The JECS has 15 regional centers (RC) nationwide (Fig. 1), and data can be compared between post-earthquake areas and the whole country; therefore, we used data from the JECS in the present study. Thus, this study aimed to follow-up on the prevalence of psychological distress after the Great East Japan Earthquake.

## Methods

### Study design

This study was a part of the JECS, which was initiated by the Ministry of the Environment in Japan as a nationwide prospective birth cohort study to investigate the association between environmental factors and children’s health and development. Women and their families participated between January 2011 and May 2014 via 15 RCs assented to the JECS (Fig. 1). All participants provided written informed consent. Institutional review boards approved the JECS protocol at the Japanese Ministry of Environment and all of the participating institutions. The Programme Office of the JECS provided several data sets for research groups in series. Each research group uses released data sets to investigate its research subject. The present analysis is based on the all-birth fixed data sets, “jecs-ag-20160424” and “allbirth_revice001,” which were released in June 2016 and October 2016, respectively. These data sets report information on 104,102 fetuses and their parents.

The Miyagi Regional Center (Miyagi RC) was selected as the disaster area, and the other “13 RCs” that had a mortality rate of 0.5 or less per 100,000 population due to the direct impact of the earthquake were selected as control areas because this study was intended to assess the impact of the disaster, especially of the tsunami. We excluded the Fukushima RC from the control area of 13 RCs because of the large impact of the disaster related to the nuclear power plant. Furthermore, Miyagi RC was divided into two groups: the *coast* area, which suffered from extensive damage from the tsunami, and the *inland* area, which had massive earthquake damage but no direct tsunami damage. Seven municipalities were included in each area: Kesennuma, Minami-Sanriku, Ishinomaki, Onagawa, Iwanuma, Watari, and Yamamoto in the *coast* area, and Osaki, Wakuya, Misato, Kami, Shikama, Kurihara, and Tome in the *inland* area. The JECS study’s primary purpose is to investigate how environmental chemicals affect children’s health prospectively; it was not constructed to observe the disaster’s effects. Therefore, data were not available from the more populated cities in the affected areas.

All data were obtained from two self-reported questionnaires; the “MT1” questionnaires were administered upon enrollment and during the maternal first trimester, and the “MT2” was administered during the second or third trimester. The targeted period was from May 2011 to April 2014, in order to be consistent with previous research [13]. In the affected areas, especially in the coastal areas, the prevalence of psychological distress was high 6 months after the earthquake [13]. Therefore, we classified this time period into six sections to observe more detailed changes. Participants were divided into 6-month groups based on the day of their response to MT2: from May 2011 to October 2011, November 2011 to April 2012, May 2012 to October 2012, November 2012 to April 2013, May 2013 to October 2013, or November 2013 to April 2014. The groups were named 2011H1, 2011H2, 2012H1, 2012H2, 2013H1, and 2013H2, respectively, in this analysis.

### Main outcome measurement

The Kessler 6-item psychological distress scale (K6) has been widely used as a screening scale for psychological distress in the general population [15, 16]. The Japanese version of the K6 was recently developed using the standard back-translation method [17]. The K6 consists of six questions with five possible responses (0–4) for each question: “none of the time” (0 points), “a little of the time” (1 point), “some of the time” (2 points), “most of the time” (3 points), and “all of the time” (4 points). The six questions were as follows, “During the last 30 days, how often have you felt the following, (1) nervous, (2) hopeless, (3) restless or fidgety, (4) so depressed that nothing could cheer you up, (5) that everything was an effort, and (6) worthless?” The range of total scores was from 0 to 24. As Kessler et al. [16] suggested, we classified women with K6 scores ≥ 13 as having psychological distress. In previous studies [13, 18–20], the Japanese version of the K6 has been used with the same cutoff point. The JECS protocol sets K6 measurements twice during pregnancy. In this study, mental distress in the second or third trimester of pregnancy was assessed using the K6 score of the MT2 questionnaire, in line with a previous report [13].

### Baseline characteristics and negative life events

The T1 questionnaire upon enrollment provides information about age, parity, marital status, body mass index (BMI) before pregnancy, family structure, feelings toward this pregnancy, and past history of mental illness. Information about family income, education level of the couple, smoking history of the couple, and maternal alcohol intake was gained from the T2 questionnaire in addition to the K6 scale. Results are shown in Tables 1 and 2.

**Table 1:** Baseline characteristics, total K6 scores, and negative life events according to period on The Japan Environment and Children Study

	2011H1	2011H2	2012H1	2012H2	2013H1	2013H2	*p* value
n	9513	12,004	13,569	13,405	14,659	13,002	
Age							0.0009
≤ 24 years	9.86	9.83	9.46	9.40	9.35	9.32	
25–34 years	63.99	63.84	62.69	62.79	62.25	61.87	
≥ 35 years	26.14	26.33	27.86	27.80	28.39	28.80	
Missing	0.01	0.00	0.00	0.01	0.01	0.01	
Parity							< 0.001
Primipara	37.60	37.70	38.29	38.65	38.90	41.16	
Multipara	58.54	59.24	58.35	58.85	58.01	57.59	
Missing	3.86	3.07	3.36	2.50	2.09	1.25	
Marital status							0.0027
Married	95.42	95.51	95.46	95.27	95.23	94.59	
Other	4.14	4.03	4.17	4.20	4.25	5.03	
Missing	0.44	0.46	0.37	0.53	0.52	0.38	
Family income							< 0.001
≤ 199 × 10^4^ JPY	5.78	5.49	5.65	5.36	5.33	5.08	
200–399 × 10^4^ JPY	32.75	32.63	32.83	32.50	31.48	30.77	
400–599 × 10^4^ JPY	31.22	31.22	29.91	31.00	31.55	31.56	
≥ 600 × 10^4^ JPY	24.02	24.34	24.70	24.29	25.16	27.07	
Missing	6.21	6.31	6.91	6.84	6.48	5.51	
Education level							
Maternal							<0.0001
Junior high school	7.72	7.49	7.57	7.18	7.39	7.31	
Senior high school	37.05	36.06	35.03	35.39	34.55	33.89	
College	54.34	55.66	56.39	56.39	57.04	57.63	
Missing	0.89	0.78	1.02	1.04	1.02	1.16	
Paternal							< 0.0001
Junior high school	4.95	4.81	5.07	5.07	4.71	4.86	
Senior high school	30.84	31.87	30.21	30.76	29.75	28.75	
College	63.79	63.05	64.29	63.77	65.12	66.02	
Missing	0.42	0.27	0.43	0.41	0.42	0.37	
Body mass index							< 0.0001
< 18.5 kg/m^2^	16.11	15.80	15.90	17.00	15.64	16.91	
18.5–24.9 kg/m^2^	72.12	72.80	73.21	71.58	72.67	72.16	
≥ 25 kg/m^2^	9.77	9.70	9.64	10.26	10.86	10.16	
Missing	2.00	1.70	1.25	1.16	0.83	0.77	
	2011H1	2011H2	2012H1	2012H2	2013H1	2013H2	*p* value
*n*	9513	12,004	13,569	13,405	14,659	13,002	
Smoking							
Maternal							0.0005
Yes	5.25	4.61	4.89	4.74	4.65	4.15	
No	94.06	94.52	94.23	94.26	94.55	95.23	
Missing	0.69	0.87	0.88	1.00	0.80	0.62	
Paternal							0.3362
Yes	45.62	45.48	45.56	44.96	44.72	45.06	
No	52.63	52.80	52.63	53.18	53.70	53.42	
Missing	1.74	1.72	1.81	1.86	1.58	1.52	
Alcohol intake							< 0.0001
Yes	3.90	3.47	3.21	2.73	2.46	2.35	
No	95.58	95.96	96.15	96.14	96.35	96.77	
Missing	0.52	0.57	0.65	1.13	1.19	0.88	
Feeling toward this pregnancy							0.9068
Uneasy	7.98	7.47	7.66	7.50	7.74	7.40	
Other	91.73	92.19	92.02	92.14	91.95	92.31	
Missing	0.29	0.33	0.32	0.36	0.31	0.29	
Past history of mental illness							0.452
Yes	7.25	7.71	7.82	7.73	7.68	8.01	
No	92.75	92.29	92.18	92.27	92.32	91.99	
Fetal number							< 0.0001
Singleton	99.21	99.83	99.31	96.49	91.69	86.42	
Multiple	0.79	0.17	0.69	3.51	8.30	13.58	
Number of times participating in JECS							< 0.0001
1	99.99	99.83	99.32	96.51	91.74	86.42	
2	0.01	0.17	0.68	3.49	8.23	13.42	
3	0.00	0.00	0.00	0.00	0.03	0.16	
Total scoring of K6 points							0.0598
≤ 4	78.10	78.87	77.57	77.64	76.92	77.40	
5–9	15.00	14.57	15.59	15.22	15.70	15.62	
10–12	3.63	3.49	3.32	3.51	3.78	3.37	
≥ 13	3.27	3.07	3.52	3.63	3.60	3.62	
	2011H1	2011H2	2012H1	2012H2	2013H1	2013H2	*p* value
*n*	9513	12,004	13,569	13,405	14,659	13,002	
Negative life events^*1^							< 0.001
Yes	43.95	45.28	42.26	42.83	41.88	41.92	
No	55.30	54.01	56.86	56.54	57.42	57.50	
Missing	0.75	0.71	0.88	0.63	0.70	0.58	
Classification of negative life events							
Bereavement							
Close blood relative	2.57	2.54	2.39	2.15	2.32	2.31	0.562
Close friend	1.59	1.51	1.02	1.04	0.91	0.80	< 0.001
Injury and illness							
Close blood relative	15.23	16.18	15.17	14.37	14.71	14.39	0.2155
Dismissal							
Self	1.43	1.27	1.16	1.10	1.03	0.96	0.0305
Husband	1.40	1.08	1.12	1.10	0.84	0.86	0.0003
Enormous debt	0.95	0.92	0.87	0.86	0.85	0.88	0.8872
Change of family structure	4.65	4.61	4.28	4.00	3.70	3.63	< 0.001
Change of residence	9.60	9.81	10.04	9.91	10.68	10.48	0.0390
Marital problems	9.93	10.25	9.77	10.50	9.69	10.16	0.2208
Divorce	0.30	0.27	0.45	0.31	0.28	0.28	0.0762

*2011H1* from May 2011 to October 2011, *2011H2* from November 2011 to April 2012, *2012H1* from May 2012 to October 2012, *2012H2* from November 2012 to April 2013, *2013H1* from May 2013 to October 2013, *2013H2* from November 2013 to April 2014

Miyagi RC (Regional Center) was combined with inland and coast

*13 RCs* the 13 regional centers other than Miyagi RC and Fukushima RC

^*1^ Negative life events in this survey refer to events within the past year at the time the survey was conducted

Regarding the feelings toward this pregnancy, “uneasy” was defined as cases in which participants felt unsure and embarrassed about their pregnancy. Moreover, past history of mental illness included reports of depression, anxiety disorders, schizophrenia, and dysautonomia, which women had experienced before pregnancy. Additionally, fetal number in this pregnancy was classified by singleton and multiple, including twins and triplets.

Previous life events affect women’s psychological condition during pregnancy and after delivery [12, 21–23]. Negative life events were defined as the experience of any of the following circumstances: bereavement of close blood relatives and friends, injury and illness of close blood relatives, unemployment of self and husband with large debts, change of family structure, change of residence, marital problems, and divorce. In this study, the data on negative life events were obtained from the T2 questionnaire.

### Statistical analyses

The prevalence rates of psychological distress in the north coastal, south coastal, and inland areas of Miyagi, as well as 13 RCs, were calculated for each year using a chi-square test, and we performed trend test each area (Table 3). Univariate analysis and logistic regression analysis were used (with 13 RCs in 2011H1 as the reference group) to compare the prevalence rates for these areas. In two models of multivariable logistic regression analyses, we calculated the adjusted odds ratio (aOR) of each area in Miyagi for psychological distress in Tables 4 and 5. Model 1 was the logistic regression analysis adjusted for all baseline characteristics (age, parity, marital status, family income, education level, alcohol intake, feeling toward this pregnancy, past history mental illness, fetal number, and number of times participating in the JECS). In model 2, we adjusted for baseline characteristics with the addition of negative life events. We performed stratified analyses to confirm the interaction between regionality and negative life events. We converted missing values to dummy variables and statistically processed them. All analyses were performed using SAS version 9.4 (SAS Institute Inc., Cary, NC, USA).

**Table 2:** Baseline characteristics, total scoring of K6 points, and negative life events according to region on The Japan Environment and Children Study

	13 RCs	Miyagi RC	*p* value	Inland	Coast	*p* value
*N*	67,882	8270		5015	3255	
Age			< 0.001			< 0.001
≤ 24 years	8.91	14.44		15.11	13.39	
25–34 years	62.64	64.35		65.42	62.70	
30–34 years	28.45	21.16		19.42	23.84	
Missing	0.00	0.05		0.04	0.06	
Parity			< 0.001			< 0.001
Primipara	38.97	38.97		38.70	39.39	
Multipara	58.21	60.04		60.34	59.57	
Missing	2.82	0.99		0.96	1.04	
Marital status			< 0.001			< 0.001
Married	95.34	94.35		94.50	94.13	
Other	4.20	5.26		5.08	5.53	
Missing	0.46	0.39		0.42	0.34	
Family income			< 0.001			< 0.001
≤ 199 × 10^4^ JPY	5.28	6.72		6.42	7.19	
200–399 × 10^4^ JPY	31.69	35.63		33.98	38.19	
400–599 × 10^4^ JPY	31.67	26.14		25.60	26.97	
≥ 600 × 10^4^ JPY	25.53	20.53		21.14	19.61	
Missing	5.84	10.97		12.86	8.05	
Education level			< 0.001			< 0.001
Maternal						
Junior high school	7.29	8.60		8.37	8.94	
Senior high school	33.24	51.45		51.15	51.92	
College	58.46	39.07		39.56	38.31	
Missing	1.01	0.88		0.92	0.83	
Paternal			< 0.001			< 0.001
Junior high school	4.84	5.48		5.40	5.59	
Senior high school	28.24	47.32		48.10	46.11	
College	66.53	46.88		46.16	47.99	
Missing	0.39	0.33		0.34	0.31	
Body mass index			< 0.001			< 0.001
< 18.5 kg/m^2^	16.50	13.98		14.06	13.86	
18.5–24.9 kg/m^2^	72.51	71.86		71.98	71.71	
≥ 25 kg/m^2^	9.61	14.14		13.94	14.44	
Missing	1.38	0.01		0.02	0.00	
	13 RCs	Miyagi RC	*p* value	Inland	Coast	*p* value
*n*	67,882	8270		5015	3255	
Smoking						
Maternal			< 0.001			< 0.001
Yes	4.50	6.25		5.94	6.73	
No	94.68	92.96		93.10	92.75	
Missing	0.82	0.79		0.96	0.52	
Paternal			< 0.001			< 0.001
Yes	43.43	59.77		59.58	60.06	
No	54.83	38.86		39.06	38.56	
Missing	1.74	1.37		1.36	1.38	
Alcohol intake			< 0.001			<0.001
Yes	3.01	2.54		2.71	2.27	
No	96.04	97.39		97.21	97.67	
Missing	0.95	0.07		0.08	0.06	
Feeling toward this pregnancy			< 0.001			< 0.001
Uneasy	7.45	8.98		8.91	9.06	
Other	92.22	90.83		90.95	90.66	
Missing	0.34	0.19		0.14	0.28	
Past history of mental illness			0.4671			0.650
Yes	7.75	7.52		7.66	7.31	
No	92.25	92.48		92.34	92.69	
Fetal number			0.1027			0.0418
Singleton	99.04	99.23		99.39	98.98	
Multiple	0.96	0.77		0.61	1.02	
Number of times participating in JECS			< 0.001			< 0.001
1	95.43	94.35		93.32	95.94	
2	4.54	5.60		6.62	4.02	
3	0.03	0.05		0.06	0.03	
Total scoring of K6 points			< 0.001			< 0.001
≤ 4	78.22	73.37		72.86	74.16	
5–9	15.08	17.25		16.94	17.74	
10–12	3.41	4.43		4.80	3.86	
≥ 13	3.29	4.95		5.41	4.23	
	13 RCs	Miyagi RC	*p* value	Inland	Coast	*p* value
*n*	67,882	8270		5015	3255	
Negative life events^*1^			< 0.001			< 0.001
Yes	42.28	48.14		45.98	51.49	
No	56.97	51.53		53.60	48.33	
Missing	0.75	0.33		0.42	0.18	
Classification of negative life events						
Bereavement						
Close blood relative	2.28	3.08	0.0862	2.37	4.15	0.082
Close friend	0.97	2.27	< 0.001	1.24	3.87	< 0.001
Injury and Illness						
Close blood relative	15.43	13.50	0.034	13.91	12.85	0.042
Dismissal						
Self	1.07	1.75	< 0.001	1.56	2.06	< 0.001
Husband	1.01	1.34	0.0051	1.12	1.69	< 0.001
Enormous debt	0.90	0.70	0.0621	0.56	0.92	0.0396
Change of family structure	3.61	8.14	<0.001	7.74	8.76	<0.001
Change of residence	10.00	11.14	0.0012	9.97	12.93	<0.001
Marital problems	10.02	10.27	0.4802	10.45	9.98	0.6161
Divorce	0.32	0.27	0.3984	0.22	0.34	0.4501

Miyagi RC (Regional Center) was combined with inland and coast

*13RCs* the 13 regional centers not including Miyagi RC and Fukushima RC

^*1^ Negative life events in this survey refer to events within the past year at the time of answering the questions

**Table 3:** Interannual change in psychological distress (K6 ≥ 13) on the coast, inland of Miyagi, and in the 13 RCs

		2011H1	2011H2	2012H1	2012H2	2013H1	2013H2	Chi-square p†	Trend p
13 RCs(*n* = 67,882)	% (*n*)	2.74 (232/8463)	2.61 (270/10346)	3.11 (375/12042)	3.14 (373/11899)	3.29 (434/13193)	3.21 (383/11939)	0.0187	0.0018
Miyagi RC (*n* = 8270)	% (*n*)	5.14 (54/1050)	4.28 (71/1658)	4.19 (64/1527)	5.05 (76/1506)	3.75 (55/1466)	4.99 (53/1063)	0.4087	0.7773
Chi-square *p*†		< 0.001	0.0001	0.0250	0.0001	0.3499	0.0020		
Inland (*n* = 5015)	% (*n*)	4.99 (35/702)	4.68 (46/983)	4.96 (46/927)	5.81 (51/878)	4.50 (38/844)	4.70 (32/681)	0.8515	0.9079
Coast (*n* = 3255)	% (*n*)	5.46 (19/348)	3.70 (25/675)	3.00 (18/600)	3.98 (25/628)	2.73 (17/622)	5.50 (21/382)	0.1265	0.8022
Chi-square *p*†		< 0.0001	0.0004	0.0086	< 0.001	0.1136	0.0067		

*2011H1* from May 2011 to October 2011, *2011H2* from November 2011 to April 2012, *2012H1* from May 2012 to October 2012, *2012H2* from November 2012 to April 2013, *2013H1* from May 2013 to October 2013, *2013H2* from November 2013 to April 2014

Miyagi RC was combined with inland and coast

13 RCs (Regional Centers): the 13 regional centers other than Miyagi RC and Fukushima RC

Chi-square *p*†: chi-square *p* values from the comparison of percentages across the time points in each site

Chi-square *p*‡: chi-square *p* values from the comparison of percentages across the sites in each time point

**Table 4:** Logistic analysis for pregnant women with psychological distress (K6 ≥ 13) in Miyagi RC and the 13 RCs

			2011H1	2011H2	2012H1	2012H2	2013H1	2013H2
	13 RCs(*n* = 67,882)	OR(95%CI)	Ref	0.95(0.80–1.14)	1.14(0.97–1.35)	1.15(0.97–1.36)	1.21(1.03–1.42)	1.18(1.00–1.39)
	Miyagi RC (*n* = 8270)	OR(95%CI)	1.92 (1.42–2.60)	1.59(1.21–2.08)	1.55(1.17–2.06)	1.89(1.45–2.46)	1.38(1.03–1.87)	1.86(1.37–2.53)
^*^Model 1	13 RCs(*n* = 67,882)	OR(95%CI)	Ref	0.96(0.80–1.15)	1.15(0.97–1.37)	1.16(0.98–1.38)	1.22(1.08–1.44)	1.21(1.02–1.43)
	Miyagi RC (*n* = 8270)	OR(95%CI)	1.65(1.21–2.2.25)	1.40(1.06–1.85)	1.34(1.00–1.78)	1.65(1.26–2.17)	1.24(0.92–1.69)	1.72(1.26–2.36)
**Model 2	13 RCs(*n* = 67,882)	OR(95%CI)	Ref	0.95(0.79–1.14)	1.18(0.99–1.40)	1.17(0.99–1.39)	1.26(1.07–1.49)	1.23(1.04–1.46)
	Miyagi RC (*n* = 8270)	OR(95%CI)	1.48(1.08–2.03)	1.25(0.94–1.65)	1.28(0.96–1.71)	1.68(1.28–2.21)	1.26(0.93–1.72)	1.80(1.32–2.47)

*2011H1* from May 2011 to October 2011, *2011H2* from November 2011 to April 2012, *2012H1* from May 2012 to October 2012, *2012H2* from November 2012 to April 2013, *2013H1* from May 2013 to October 2013, *2013H2* from November 2013 to April 2014

*OR* odds ratio, *CI* confidence interval. Miyagi RC was combined with inland and coast

13 RCs (Regional Centers): the 13 regional centers other than Miyagi RC and Fukushima RC

*Model 1: adjusted for baseline characters: age, parity, marital status, family income, education level of couple, body mass index, smoking status of couple, alcohol intake, feeling toward this pregnancy, past history of mental illness, fetal number, and number of times participating in the JECS

**Model 2: model 1 with negative life events added. Negative life events in this survey refer to events within the past year at the time of answering the questions

## Results

**Fig. 2:**
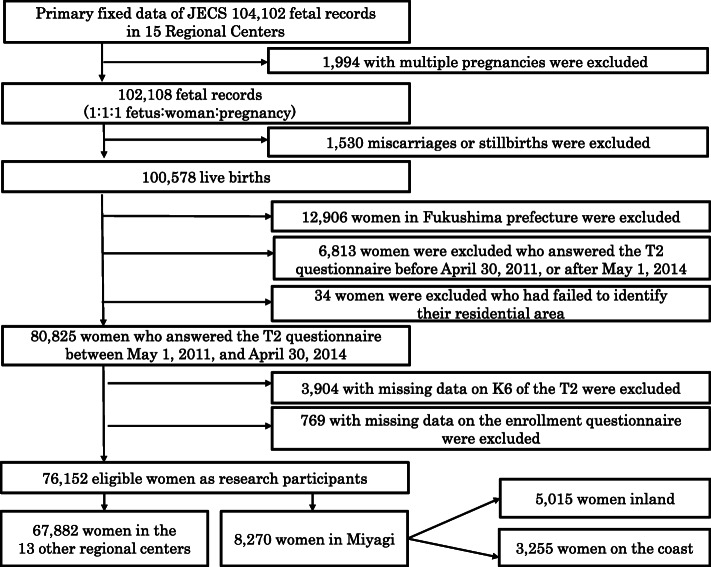
Participants flow diagram

The participant flow diagram is shown in Fig. 2. The fixed data of the JECS include 104,102 fetuses and their mothers from all RC. Of those, 1994 with multiples pregnancies were excluded. Among the remaining, 100,578 had given birth, and the other 1530 had miscarriages or stillbirths. Within the study period, the T2 questionnaire was obtained from 80,825 women. Women who did not provide information on the enrollment questionnaire (*n* = 769) and with missing data on the K6 (*n* = 3904) were excluded. In the remaining 76,152 eligible women, 8270 were from Miyagi RC, including 5015 pairs from the *inland* area and 3255 pairs from the *coast* area. Table 2 shows baseline characteristics, total scoring of K6 points, and negative life events according to region on the Japan Environment and Children Study.

**Table 5:** Logistic analysis for pregnant women with psychological distress (K6 ≥ 13) on the coast, inland of Miyagi, and in the 13 RCs

			2011H1	2011H2	2012H1	2012H2	2013H1	2013H2
	13 RCs(*n* = 67,882)	OR(95%CI)	ref	0.95(0.80–1.14)	1.14(0.97–1.35)	1.15(0.97–1.36)	1.21(1.03–1.42)	1.18(1.00–1.39)
	Inland (*n* = 5015)	OR(95%CI)	1.86(1.29–2.68)	1.74(1.26–2.41)	1.85(1.34–2.56)	2.19(1.60–2.99)	1.67(1.18–2.38)	1.75(1.20–2.55)
	Coast (*n* = 3255)	OR(95%CI)	2.05(1.27–3.31)	1.37(0.90–2.08)	1.10 (0.67–1.79)	1.47(0.97–2.24)	1.00(0.61–1.64)	2.06(1.30–3.27)
*Model 1	13 RCs(*n* = 67,882)	OR(95%CI)	ref	0.96(0.80–1.15)	1.15(0.97–1.37)	1.16(0.98–1.38)	1.22(1.08–1.44)	1.21(1.02–1.43)
	Inland (*n* = 5015)	OR(95%CI)	1.60(1.10–2.33)	1.55(1.11–2.16)	1.55(1.11–2.16)	1.91(1.39–2.64)	1.54(1.08–2.20)	1.58(1.07–2.33)
	Coast (*n* = 3255)	OR(95%CI)	1.75(1.07–2.87)	1.19(0.78–1.83)	0.99(0.60–1.62)	1.30(0.84–2.00)	0.87(0.52–1.44)	1.99 (1.25–3.18)
**Model 2	13 RCs(*n* = 67,882)	OR(95%CI)	ref	0.95(0.79–1.14)	1.18(0.99–1.40)	1.17(0.99–1.39)	1.26(1.07–1.49)	1.23(1.04–1.46)
	Inland (*n* = 5015)	OR(95%CI)	1.52(1.04–2.22)	1.45(1.04–2.02)	1.51(1.08–2.11)	1.97(1.43–2.73)	1.58(1.10–2.26)	1.61(1.09–2.39)
	Coast (*n* = 3255)	OR(95%CI)	1.41(0.860–2.306)	1.00(0.65–1.53)	0.92(0.56–1.52)	1.30(0.84–2.00)	0.87(0.53–1.46)	2.18(1.37–3.49)

*2011H1* from May 2011 to October 2011, *2011H2* from November 2011 to April 2012, *2012H1* from May 2012 to October 2012, *2012H2* from November 2012 to April 2013, *2013H1* from May 2013 to October 2013, *2013H2* from November 2013 to April 2014.

*OR* odds ratio, *CI* confidence interval. Miyagi RC was combined with inland and coast

13 RCs (Regional Centers): the 13 regional centers without Miyagi RC and Fukushima RC

*Model 1: adjusted for baseline characters: age, parity, marital status, family income, education level of couple, body mass index, smoking status of couple, alcohol intake, feeling toward this pregnancy, past history of mental illness, fetal number, and number of times participating in the JECS

**Model 2: model 1 with negative life events added. Negative life events in this survey refer to events within the past year at the time of answering the questions.

Table 3 shows inter-semiannual changes in K6 ≥ 13 for different areas. More women in Miyagi RC suffered psychological distress compared to the other 13 RCs. In the inland area, the prevalence of psychological distress was consistently higher than that in the 13 RCs after the disaster. In the 13 RCs, the prevalence of psychological distress significantly increased over the years (trend *p* = 0.002).

In the multivariate logistic analysis, the Miyagi RC had a consistently higher risk of psychological distress compared to the 13 RCs in 2011H1: OR 1.38 (95% CI, 1.03–1.87) to 1.92 (95% CI, 1.42–2.60) (Table 4). When the Miyagi RC was subdivided into inland and coast (Table 5), the inland area had a consistently higher risk of psychological distress compared to the 13 RCs in 2011H1: OR 1.67 (95% CI 1.18–2.38) to 2.19 (95% CI, 1.60–2.99). When comparing the 13RCs and Miyagi RC in a multivariable analysis after adjusting for possible confounding factors (*Model 1*), the *inland* area had significantly higher risks for psychological distress (K6 ≥ 13) than the 13 RCs in 2011H’: OR 1.54 (95% CI 1.08–2.20) to 1.91 (95% CI 1.39–2.64). Even after the further adjustment for negative life events, significantly higher risks remained; *Model 2*: OR 1.45 (95%CI 1.04–2.02) to 1.97 (95% CI 1.43–2.73). On the other hand, the further adjustment with negative life events removed the significantly high risk for psychological distress in 2011H1—Crude: OR 2.05 (95% CI 1.27–3.31), *Model 1:* OR 1.75 (95% CI 1.07–2.87), *Model 2:* OR 1.41 (95% CI 0.86–2.31)—on the coast of Miyagi.

Even for the pregnant women in the 13RCs, the risk of psychological distress (K6 ≥ 13) increased from 2011 and reached significance after 2013H1 as compared to the “13 RCs 2011H1”; *Model 1*; OR 1.22 (95% CI 1.08–1.44) in 2013H1, and OR 1.21 (95% CI 1.02–1.43) in 2013H2. Even after the further adjustment for negative life events, significantly higher risks remained; *Model 2*: OR 1.26 (95% CI 1.07–1.49) in 2013H1, and OR 1.23 (95% CI 1.04–1.46) in 2013H2.

## Discussion

The literature on changes in the prevalence of psychological distress due to disaster on pregnancy and the postpartum period is limited. However, natural disasters have been reported to affect mothers’ psychological states and to increase depressive symptoms during and after pregnancy [4, 24, 25]. We previously mentioned that the prevalence of psychological distress among pregnant women was high in Miyagi Prefecture [13]. Further, the risk of psychological distress remained when they had experienced negative life events [13]. In a cross-sectional study conducted in Miyagi Prefecture immediately after the disaster, 21.5% of pregnant women scored 9 points or more (indicating postpartum depression) on the Edinburgh Postpartum Depression Questionnaire. This percentage is significantly higher than the usual 10–15% (Arima, 2013). The pregnant women who were affected by the disaster (due to being evacuated or having no work) had a high risk of postpartum depression [26].

As for the *inland* area, the pregnant women had a significantly higher risk for psychological distress compared to the 13 RCs, 2011H1 after adjustment for baseline characteristics. Moreover, even after further adjusting for negative life events, the risk of psychological distress remained. According to Miyagi Prefecture Earthquake Reconstruction and Planning Department Statistics Division, 2015, the population change rate in 2010–2015 as compared to 2005–2010 was − 7 points and + 1.5 points in the *coast* area and *inland* area, respectively. Meanwhile, a previous study has reported that relocation after a disaster is associated with a risk for depression [27]. Further, Hansel et al. [28] observed an increase in posttraumatic stress symptoms in people relocated from the disaster area and stated that support was needed not only in disaster areas but also in resettled areas. There was a possibility that the focus on victims was concentrated in coastal areas, whereas the focusing might be inadequate for relatively less damaged inland areas with many migrants (during the progress of reconstruction). According to the summary by the Reconstruction Agency, many financial resources were allocated to reconstruction assistance after the Great East Japan Earthquake, but in Miyagi, such assistance was concentrated in the coastal areas [29]. It has been reported that those who received a large amount of social support at the time of the Great East Japan Earthquake saw substantial improvements in psychological distress [30]. More extensive care is required, not only in the directly affected area but also in the surrounding areas—especially in the places to which the affected people moved. There might also be a need to pay more attention to the surrounding regions that received moderate damage.

Most of the disaster prevention countermeasures were led by men in Japan. In this context, inadequate attention to gender issues was pointed out in the case of the Great East Japan Earthquake. Moreover, Domoto et al. [31] discussed the importance of gender sensitivity in disaster risk reduction. Countermeasures, such as securing women's privacy and safety at shelters, care during pregnancy, gender-based care, and appropriate care for disabled people should thus be considered. More specifically, Yoshida et al. [32] stated that there is an urgent need to investigate the construction of the system focusing on the mother and the child in terms of the resilience surrounding the community at the time of disaster. Moreover, depression and anxiety symptoms during pregnancy are associated with attentional disorders in childhood, intelligence quotient decline in school children, and internalization and externalization problem behaviors [33, 34]. Stress during pregnancy, such as disaster, wars, and intimate partner violence, is also considered to influence children’s long-term outcomes [35, 36]. Thus, it is crucial to maintain an environment where pregnant women and postpartum women can take care of their children without anxiety as much as possible.

In the present study, the prevalence of pregnant women with psychological distress in the 13 RCs was 2.74% in 2011H1, which is almost consistent with the previous data from a Japanese nationwide survey in 2007 [37]. Subsequently, the incidence of psychological distress during pregnancy significantly increased in the 13RCs. Pregnant women with psychological distress had a high risk of having problems with child-rearing and attachment formation [38]. Thereby, the Japan Society of Obstetrics and Gynecology [39] advocated the establishment of a system for the early detection and linking for psychosocial high-risk pregnant women to an appropriate support (maternal mental health care manual). In this context, the risk factors for depression during pregnancy include anxiety during pregnancy, life events, past depression history, lack of social support, and unwanted pregnancy [40]. In addition to a lack of support from a spouse or other life events during pregnancy, psychological distress is a risk factor for postpartum depression [23, 41]. The increasing incidence of psychological distress during pregnancy is an essential issue for Japan. Thus, appropriate continuous support might be necessary.

Pregnant women in *coast* areas who participated in 2013H2 showed a significantly higher risk of depression than those from the 13 RCs who participated in 2011H1; however, the result is different from other semi-annual trends. The number of participants decreased in 2013H2 due to new recruitment for The Birth and Three Generation Cohort Study (the Birth Three Cohort Study [42]). The participants in the *inland* area and the *coast* area in 2013H2 were 5.2% and 2.9%, and the proportion of participants was smaller than that of the other periods (6.9% and 4.6%). Consequently, it is necessary to wait for the results of new cohort studies in the future to determine whether the increase in psychological distress is due to environmental factors or the decrease in the number of participants.

### Limitations

This study has several limitations. First, comparisons before and after the Great East Japan Earthquake were not possible because pre-disaster data were not available. Second, there is a possibility that the circumstances surrounding pregnant women change not only over time, but also in different groups. There is an item in the JECS questionnaire regarding a change of residence within 1 year prior to answering, but it was difficult to consider all of the relocations since the disaster. Thus, we could not completely adjust for the relocations of people between regions. Third, participation in JECS was not compulsory; thus, there is a limitation regarding regional representativeness. Additionally, it might be challenging for pregnant women who lived regions of severe damage immediately after the disaster to participate in this study. However, JECS contacted as many expecting mothers who reside in the study areas as possible, and the recruitment rate is targeted to be more than 50% of all eligible mothers [43]. Despite these limitations, the present study is a unique report showing changes over time in the prevalence of K6 ≥ 13 in pregnant women after a large-scale disaster.

## Conclusion

Three years after the Great East Japan Earthquake, the prevalence of pregnant women with psychological distress has been maintained in the Miyagi Prefecture. Notably, in *inland* areas, the prevalence of mental illness was consistently higher than the prevalence in the other 13RCs, while the risk of psychological distress in the 13 RCs was significantly increased. Thus, continuous support, including in the surrounding areas, is necessary for the future.

## Data Availability

All data generated and analyzed during this step of the study are included in this published article.

## Abbreviations

aOR: Adjusted odds ratio
BMI: Body mass index
CI: Confidence interval
DSM: Diagnostic and Statistical Manual of Mental Disorders
JECS: the Japan Environment and Children’s Study
K6: Kessler 6-item psychological distress scale
NLE: Negative life events
OR: Odds ratio
RC: Regional Centers
SD: Standard deviation

## References

[1] Fire and Disaster Management Agency of the Ministry of Internal Affairs and Communications, 2019. https://www.fdma.go.jp/disaster/higashinihon/items/159.pdf. (in Japanese), Accessed 30 Dec 2020.

[2] Miyagi Prefectural Government. About the situation such as earthquake damage and evacuation situation of the Great East Japan Earthquake, 2019.https://www.pref.miyagi.jp/uploaded/attachment/742194.pdf. (in Japanese). Accessed 10 Feb 2021.

[3] Hayes GP, Myers EK, Dewey JW, Briggs RW, Earle PS, Benz HM, et al. Tectonic summaries of magnitude 7 and greater earthquakes from 2000 to 2015: U.S. Geological Survey Open-File Report 2016–1192, 148. 2017. 10.3133/ofr20161192.

[4] Dong X, Qu Z, Liu F, Jiang X, Wang Y, Chui CH (2013). Depression and its risk factors among pregnant women in 2008 Sichuan earthquake area and non-earthquake struck area in China. J Affect Disord..

[5] Harville EW, Xiong X, Pridjian G, Elkind-Hirsch K, Buekens P (2009). Post- partum mental health after Hurricane Katrina: a cohort study. BMC Pregnancy Childbirth..

[6] Field T (2011). Prenatal depression effects on early development: a review. Infant Behav Dev..

[7] Kitamura T, Ohashi Y, Kita S, Haruna M, Kubo R (2013). Depressive mood, bonding failure, and abusive parenting among mothers with three-month-old babies in a Japanese community. Open J Psychiatry..

[8] Mulder EJ, Robles de Medina PG, Huizink AC, Van den Bergh BR, Buitelaar JK, Visser GH (2002). Prenatal maternal stress: effects on pregnancy and the (unborn) child. Early Hum Dev..

[9] Straub H, Adams M, Kim JJ, Silver RK (2012). Antenatal depressive symptoms increase the likelihood of preterm birth. Am J Obstet Gynecol.

[10] O’Connor TG, Ben-Shlomo Y, Heron J, Golding J, Adams D, Glover V (2005). Prenatal anxiety predicts individual differences in cortisol in pre-adolescent children. Biol Psychiatry..

[11] Takeda S (2016). The challenge to maternal death "zero". Japan J Obstet Gynecol..

[12] Kitamura T, Yoshida K, Okano T, Kinoshita K, Hayashi M, Toyoda N (2006). Multicentre prospective study of perinatal depression in Japan: incidence and correlates of antenatal and postnatal depression. Arch Womens Ment Health..

[13] Watanabe Z, Iwama N, Nishigori H, Nishigori T, Mizuno S, Sakurai K (2016). Psychological distress during pregnancy in Miyagi after the Great East Japan Earthquake: The Japan Environment and Children’s Study. J Affect Disord..

[14] Michikawa T, Nitta H, Nakayama F, Yamazaki S, Isobe T, Tamura K (2018). Baseline profile of participants in the Japan Environment and Children`s Study (JECS). J Epidemiol..

[15] Kessler RC, Andrews G, Colpe LJ, Hiripi E, Mroczek DK, Normand SL (2002). Short screening scales to monitor population prevalences and trends in non-specific psychological distress. Psychol Med..

[16] Kessler RC, Barker PR, Colpe LJ, Epstein JF, Gfroerer JC, Hiripi E (2003). Screening for serious mental illness in the general population. Arch Gen Psychiatry..

[17] Furukawa TA, Kawakami N, Saitoh M, Ono Y, Nakane Y, Nakamura Y (2008). The performance of the Japanese version of the K6 and K10 in the World Mental Health Survey Japan. Int J Methods Psychiatr Res..

[18] Hayasaka K, Tomata Y, Aida J, Watanabe T, Kakizaki M, Tsuji I (2013). Tooth loss and mortality in elderly Japanese adults: effect of oral care. J Am Geriatr Soc..

[19] Hozawa A, Kuriyama S, Nakaya N, Ohmori-Matsuda K, Kakizaki M, Sone T (2009). Green tea consumption is associated with lower psychological distress in a general population: the Ohsaki Cohort 2006 Study. Am J Clin Nutr..

[20] Nakaya N, Kogure M, Saito-Nakaya K, Tomata Y, Sone T, Kakizaki M, Tsuji I (2014). The association between self-reported history of physical diseases and psychological distress in a community-dwelling Japanese population: the Ohsaki Cohort 2006 Study. Eur J Public Health..

[21] Beck CT (2001). Predictors of postpartum depression: an update. Nurs Res..

[22] Leigh B, Milgrom J (2008). Risk factors for antenatal depression, postnatal de- pression and parenting stress. BMC Psychiatry..

[23] Robertson E, Grace S, Wallington T, Steward DE (2004). Antenatal risk factors for postpartum depression: a synthesis of recent literature. Gen Hosp Psychiatry..

[24] Chang HL, Chang TC, Lin TY, Kuo SS (2002). Psychiatric morbidity and pregnancy outcome in a disaster area of Taiwan 921 earthquake. Psychiat Clin Neurosci..

[25] Xiong X, Harville EW, Mattison DR, Elkind-Hirsch K, Pridjian G, Buekens P (2010). Hurricane Katrina experience and the risk of post-traumatic stress dis- order and depression among pregnant women. Am J Disaster Med..

[26] Arima T (2013). Genomic cohort study on mental health and fetal and neonatal health impact of Miyagi pregnancy after large-scale disaster. Daiwa Securities Health Foundation Research Publications (Japanese)..

[27] Najarian LM, Goenjian AK, Pelcovitz D, Mandel F, Najarian B (2001). The effect of relocation after a natural disaster. J Trauma Stress..

[28] Hansel TC, Osofsky JD, Osofsky HJ, Friedrich P (2013). The effect of long-term relocation on child and adolescent survivors of Hurricane Katrina. J Trauma Stress..

[29] Miyagi Prefecture Official Website. Progress of reconstruction, 2018. https://www.pref.miyagi.jp/uploaded/attachment/662290.pdf. Accessed 30 Dec 2020.

[30] Tsuchiya N, Nakaya N, Nakamura T, Narita A, Kogure M, Aida J, Tsuji I, Hozawa A, Tomita H (2016). Impact of social capital on psychological distress and interaction with house destruction and displacement after the Great East Japan Earthquake of 2011. Psychiat Clin Neurosci..

[31] Domoto A, Ohara M, Reiko A, Hara H, Amano K. Japan women’s network for disaster risk reduction (2013). https://www.gdnonline.org/resources/JapanWomensNetwork2013.pdf. Accessed 30 December 2020.

[32] Yoshida H, Kato N, Yokoyama T (2014). Trends in Maternal and Child Health (MCH) research in Japan: Here and now, and beyond. J Natl Inst Public Health..

[33] Evans J, Melotti R, Heron J, Ramchandani P, Wiles N, Murray L, Stein A (2012). The timing of maternal depressive symptoms and child cognitive development: a longitudinal study. J Child Psychol Psychiatry..

[34] Van Batenburg-Eddes T, Brion MJ, Henrichs J, Jaddoe VW, Hofman A, Verhulst FC (2013). Parental depressive and anxiety symptoms during pregnancy and attention problems in children: a cross-cohort consistency study. J Child Psychol Psychiatry..

[35] Gentile S (2017). Untreated depression during pregnancy: short- and long-term effects in offspring. A systematic review. Neuroscience..

[36] Jarde A, Morais M, Kingston D, Giallo R, MacQueen GM, Giglia L (2016). Neonatal outcomes in women with untreated antenatal depression compared with women without depression: a systematic review and meta-analysis. JAMA Psychiatry..

[37] Kawakami N, Furukawa T. Prevalence and related factors of psychological state in K6 scales in nationwide survey. 2006 Health and Labor Sciences Research Grant (Statistical Information Advanced Utilization Research Project) Statistical information on national health status Research on the examination of the system that grasps and analyzes from the household side, 2007. Shared Research Book 13-21.

[38] Ohoka H, Koude T, Goto S (2015). Effects of maternal depression on pregnancy and maternal attachment during pregnancy. Japanese J Neuropsychol..

[39] Japan Society of Obstetrics and Gynecology. Maternal mental health care manual, 23–56. http://www.jaog.or.jp/wp/wp-content/uploads/2017/11/jaogmental_L.pdf. Accessed 30 December 2020.

[40] Lancaster CA, Gold KJ, Flynn HA, Yoo H, Marcus SM, Davis MM (2010). Risk factors for depressive symptoms during pregnancy: a systematic review. Am J Obstet Gynecol..

[41] Milgrom J, Gemmill AW, Bilszta JL, Hayes B, Barnett B, Brooks J (2008). Antenatal risk factors for postnatal depression: a large prospective study. J Affect Disord..

[42] Kuriyama S, Metoki H, Kikuya M (2019). Cohort Profile: Tohoku Medical MegabankProject Birth and Three Generation Cohort Study (TMM BirThree Cohort Study): rationale, progress and perspective. Int J Epidemiol..

[43] Kawamoto T, Nitta H, Murata K, Toda E, Tsukamoto N, Hasegawa M (2014). Rationale and study design of the Japan environment and children's study (JECS). BMC Public Health..

